# Evaluation of an automated reader for disc diffusion antimicrobial susceptibility testing in a routine microbiology laboratory

**DOI:** 10.1371/journal.pone.0347932

**Published:** 2026-05-08

**Authors:** Patricia Orlandi Barth, Xander Cortez Pinzas, Vitória Torrescasana Teixeira, Larissa Lutz, Caroline Collioni Constante, Bruno Rodriguez Tondin, André Frotta Müller, Danton Pereira da Silva Junior, Paulo Roberto Stefani Sanches, Afonso Luís Barth

**Affiliations:** 1 Laboratório de Pesquisa em Resistência Antimicrobiana (LABRESIS) - Hospital de Clínicas de Porto Alegre, RS, Brasil; 2 Serviço de Pesquisa e Desenvolvimento em Engenharia Biomédica - Hospital de Clínicas de Porto Alegre, RS, Brasil; 3 Serviço de Diagnóstico Laboratorial - Unidade de Microbiologia - Hospital de Clínicas de Porto Alegre, RS, Brasil; Tribhuvan University, NEPAL

## Abstract

Antimicrobial susceptibility testing (AST) is essential for determining the *in vitro* activity of antimicrobial agents against specific microorganisms. Reliable data from AST is needed for the detection of resistance mechanisms which are crucial to ensure appropriate treatment of patients in healthcare settings or in the community. Automated AST readers of the inhibition zone provide valuable tools for standardizing the testing process in microbiology laboratories, enabling faster and more reliable interpretation of results. The aim of this study was to evaluate AntibiHórus, an in-house developed automated disc diffusion AST reader, by comparing its performance against manual reading of the inhibition zones using a ruler. A total of 500 ASTs, including both Gram-negative and Gram-positive bacteria, were evaluated using 22 different antimicrobial discs, resulting in 4,709 inhibition zone measurements. Manual readings with a ruler were compared with the automated readings from AntibiHórus. The overall categorical agreement (CA) between AntibiHórus and manual reading was 96.86%, with minimal error rates, 2.08% for Very Major Errors (VME), 0.30% for Major Errors (ME), and 0.76% for Minor Errors (MiE). Statistical analysis showed a strong correlation between methods (Pearson correlation coefficient R ≥ .95). The findings demonstrate that AntibiHórus is a reliable tool for routine microbiology laboratory work, enhancing the efficiency, standardization, and accuracy of AST analysis by automating inhibition zone measurement and interpretation.

## 1. Introduction

Antimicrobial susceptibility testing (AST) is a technique used to assess the *in vitro* activity of an antibiotic against a known bacterium. Data from AST is very useful to avoid clinical failure and ensure the correct therapy is administered to patients [1]. There are several methods for performing AST, which can be categorized into quantitative techniques (such as broth microdilution, agar dilution, and agar gradient diffusion strips) that provide numerical results, and qualitative methods (such as disc-diffusion), which categorize bacteria into susceptible, susceptible dose-dependent (intermediate) or resistant categories based on interpretive criteria [1–3].

Disc diffusion, originally developed by William Kirby and Alfred Bauer (Kirby–Bauer method) remains one of the most widely used methods due to its simplicity, ease of execution, reproducibility, and cost-effectiveness. Additionally, this method enables simultaneous testing of multiple antimicrobial agents and is often the first choice for validating new antimicrobials in clinical practice [4,5]. The procedure is straightforward: a standardized inoculum of the bacteria (typically 0.5 McFarland) is applied onto a Mueller-Hinton agar plate, followed by placement of antimicrobial-impregnated discs on the agar surface. The plates are then incubated for 16–18 hours at 35ºC ± 2ºC. After incubation, inhibition zones are measured with a caliper or ruler and compared to established breakpoints approved by guidelines, allowing the determination of whether the tested antimicrobial is capable of inhibiting the bacteria *in vitro*, with the potential for *in vivo* efficacy [3,6].

Despite the advantages of this technique, it requires approximately 16–18 hours to generate results, which can delay the administration of antibiotics. Delays are particularly concerning when dealing with resistant microorganisms, as they are associated with poorer outcomes, including prolonged hospital stays, increased healthcare costs, and higher mortality rates [7]. To address this challenge, automated AST systems can generate reliable results which would help the use of effective antimicrobial therapy, potentially improving patient outcomes [8]. Several commercial systems (e.g., SIRscan, ADAGIO, and WASPLab® Radian) have been evaluated in recent studies, showing high categorical agreement compared to manual reading and the potential for integration into automated laboratory workflows. However, these systems are often associated with high acquisition and maintenance costs, reliance on proprietary software, and limited accessibility in resource-constrained settings [9–12].

In response to this need, we have developed “AntibiHórus”, an automated disc-diffusion AST reader designed for routine use in microbiology laboratories. This system is capable of measuring and interpreting antimicrobial inhibition zones in a more standardized manner, minimizing human error, and accelerating AST interpretation and report release to clinicians. The primary objective of this study was to evaluate the performance of AntibiHórus by comparing its measurements and interpretations with conventional manual readings.

## 2. Materials and methods

### 2.1. AntibiHórus device

**Fig 1:**
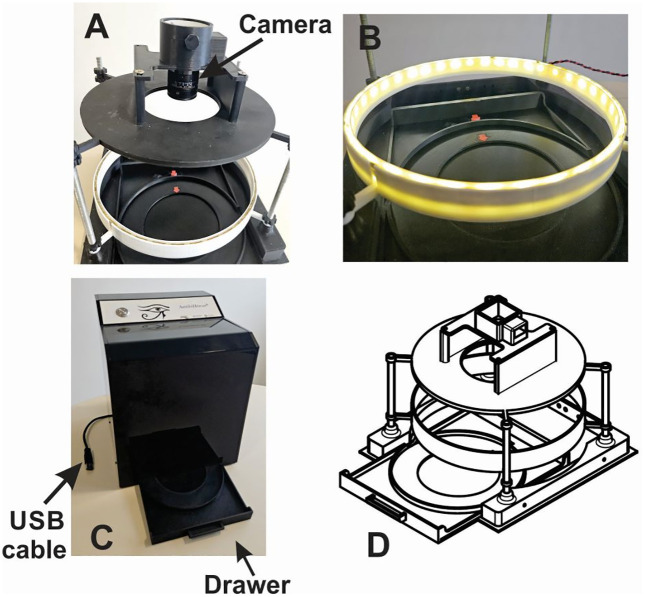
a) Visualization of the camera on the device, b) LED strip holders, c) Assembled device overview, d) Three dimensional model.

AntibiHórus was equipped with a Basler puA2500-14um camera (Ahrensburg, Germany) designed for industrial vision applications, featuring a high-resolution 5-megapixel sensor (2592 x 1944 pixels). The camera was positioned inside a light-controlled chamber (Fig 1a) to ensure consistent imaging conditions. For optimal illumination, a concentric white LED strip was installed surrounding the Petri dish position (Fig 1b), providing uniform lighting essential for accurate inhibition zone measurement. To ensure precise positioning of culture plates during analysis, we implemented a drawer system that maintained the Petri dish in a fixed position relative to the camera (Fig 1c). This component also housed the USB connection interface linking the device to the computer workstation. The entire device architecture was first developed as a three-dimensional digital model, which was subsequently fabricated using additive manufacturing technology (3D printing) to create the functional prototype shown in Fig 1d.

**Fig 2:**
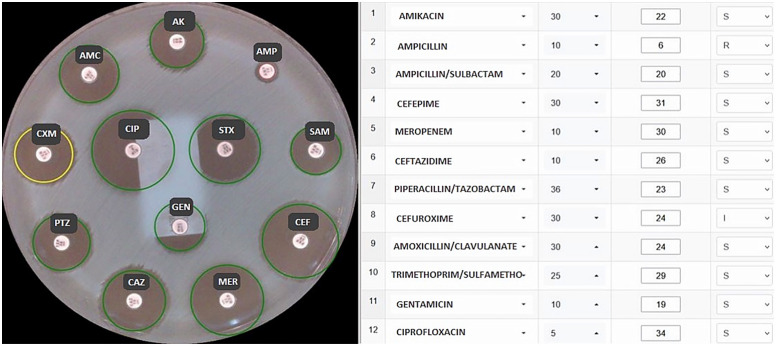
Measurement of halos (diameters) of inhibition and interpretation of results by the AntibiHórus software. S = Susceptible standard dosing, I = Susceptible increased exposure and R = Resistant. Values in the third column indicate the antibiotic concentration per disc (µg), while values in the fourth column represent the inhibition zone diameter measured in millimeters (mm).

The AntibiHóru*s* software captures high-resolution images of the agar plates, processes them using a specialized algorithm to identify disc positions and inhibition zone boundaries, precisely measures zone diameters, and automatically interprets results based on established breakpoints for each antimicrobial agent (Fig 2). In order to determine the halo diameter, the antibiotic disc positions were first accurately located within the image using the Hough Transform [13]. This provided a crucial spatial reference for the subsequent measurement phase. To overcome complexities such as variable halo shapes and lighting conditions, the image was then segmented. We employed the MiniBatchKMeans algorithm [14] to cluster the image pixels into three distinct classes: bacteria, the antibiotic disc, and the inhibition halo. Finally, the halo diameter for each antibiotic was estimated by applying the Radial Profile Analysis Algorithm (RPAA) [15] to the segmented regions centered on the discs.

### 2.2. Selection of Isolates

The study was conducted at the Microbiology Laboratory of Hospital de Clínicas de Porto Alegre (HCPA), an 860-bed tertiary care hospital in southern Brazil. Bacterial isolates subjected to AST with at least eight antibiotic discs (Mueller–Hinton agar, 180 mm) between May 2023 and March 2024 were conveniently selected for comparison between methods. All isolates were identified using Matrix Assisted Laser Desorption Ionization Time of Flight (MALDI-TOF, Biomerieux®). Additional information regarding the isolates and their susceptibility profiles is provided in Supplementary Materials 1–5.

### 2.3. Comparison of Antibihórus versus manual reading

The ASTs followed the procedures outlined by the European Committee on Antimicrobial Susceptibility Testing (EUCAST) standard guidelines [3]. Agar Mueller-Hinton plates (180 mm) containing between 8 and 12 antibiotic discs (Oxoid®) were analyzed. A total of 500 isolates, including both Gram-negative and Gram-positive bacteria, were tested with 22 antibiotics.

The ASTs were divided into five templates routinely used by the laboratory (Table 1). These templates are designated by internal codes (TU, B, C + , NF, TH), which do not represent acronyms but are internal codes adopted by the laboratory to select the antibiotics which are routinely applied to specific groups of microorganisms and clinical specimens. The templates are defined as follows:

**Table 1:** 500 AST’s evaluated into five templates. AMC = amoxicillin/clavulanate; GEN = gentamicin, PTZ = piperacillin/tazobactam; NIT = nitrofurantoin; NOR=norfloxacin; SAM = ampicillin/sulbactam; STX = trimethoprim/sulfamethoxazole; AK = Amikacin; MER = Meropenem; CXM = Cefuroxime; CAZ = Ceftazidime; CEF = Cefepime; CIP = Ciprofloxacin; AMP = Ampicillin; CLI = clindamycin; ERY = Erythromycin; DOX = Doxycycline; RIF = Rifampicin; FOX = Cefoxitin; LEV = Levofloxacin; AZT = Aztreonam; TOB = Tobramycin.

Template						
Antibiotic	TU *(n = 175)*	B *(n = 77)*	C+ *(n = 141)*	NF *(n = 68)*	TH *(n = 39)*	Total Halos
AMC	x	x			x	291
GEN	x	x	x	x	x	500
PTZ	x	x		x	x	359
NIT	x					175
NOR	x					175
SAM	x	x		x		320
STX	x	x	x	x	x	500
AK	x	x		x	x	359
MER	x	x		x	x	359
CXM	x	x				252
CAZ	x	x		x	x	359
CEF	x	x		x	x	359
CIP		x		x	x	184
AMP		x				77
CLI			x			141
ERY			x			141
DOX			x			141
RIF			x			141
FOX			x			141
LEV			x			141
AZT				x		68
TOB				x		68

- TU: Isolates of *Enterobacterales* obtained from urinary tract infections with 12 antibiotics.
- B: Isolates of *Enterobacterales* from other clinical samples with 12 antibiotics.
- C + : Isolates of *Staphylococcus* species from a variety of clinical samples with 8 antibiotics.
- NF: Isolates of Gram-negative rods Non-fermenters from a variety of clinical samples with 11 antibiotics.
- TH: Isolates of *Enterobacterales* from bloodstream infections with 9 antibiotics.

The agar plates were read manually using a ruler (reference method) and also automatically using AntibiHórus, at the same time, both after 16–18 hours of incubation. Although the system allows manual adjustment of inhibition zone measurements, we maintained all original automated readings unmodified throughout the study in order to evaluate the performance of the system without human intervention. The interpretation of AST results was performed according to EUCAST guidelines [3].

### 2.4. Data analysis

Comparison of results were classified according to the International Organization for Standardization as Categorical Agreement (CA) which indicates the number of isolates grouped in the same susceptibility category (susceptible or resistant) in both methods. Results of non agreement were classified as Very Major Errors (VME) when isolates were considered susceptible by the AntibiHórus and resistant by the manual reading, and as Major Errors (ME) when isolates were categorized as resistant by the AntibiHórus and susceptible in manual reading. Isolates categorized as resistant or susceptible by AntibiHór*us* and intermediate by manual reading were categorized as Minor Errors (MiE) [16]. Also, the means of Pearson correlation were calculated for each template.

The ethical approval was given by Porto Alegre Clinics Hospital Ethics Committee under the number CAAE 79471617000005327, and it was exempted from the consent form by the ethics committee as the study did not involve human beings, only bacterial isolates.

## 3. Results

**Fig 3:**
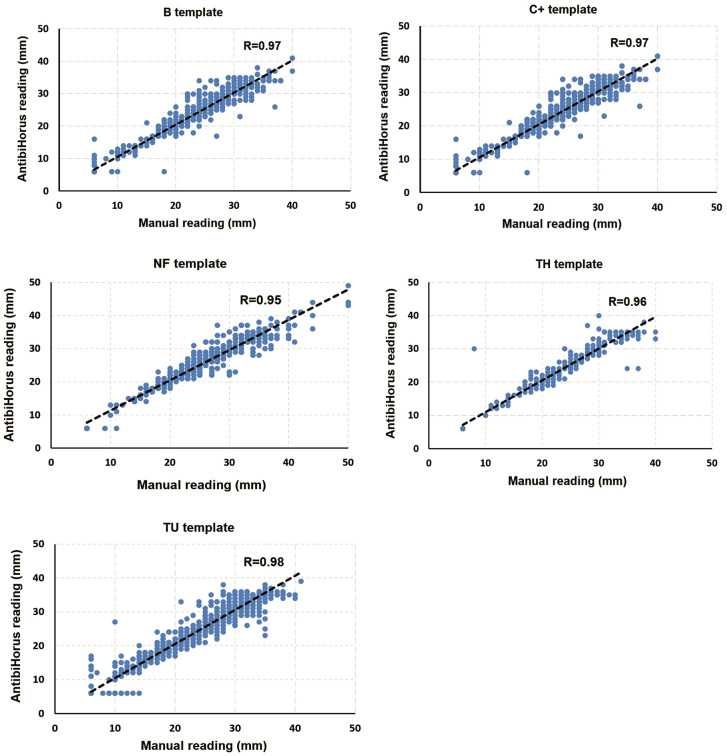
Pearson correlation between manual readings (reference method) and AntibiHórus measurements of the AST by the disc diffusion for each template.

A total of 500 ASTs of 39 bacterial species were submitted to both methods and 4,709 halos (diameters) of inhibition were analyzed. The overall CA was 96.86%, with 2.08% of VME, 0.30% of ME and 0.76% of MiE (Table 2). The overall comparison between the manual readings (reference) with the readings by AntibiHorus yielded a Pearson correlation coefficient between R = 0.95 and R = 0.98 (Fig 3). The comparison between the methods for each isolate is submitted in Supplementary Material.

**Table 2:** Errors of concordance between manual and automated (AntibiHórus) readings of the AST. VME: Very Major Error; ME: Major Error; MiE: Minor Error.

Template	Halos	VME	ME	MiE
B	801	4	2	10
C+	1121	2	2	4
TU	1938	17	4	10
NF	506	2	0	10
TH	343	0	1	2
Total	4709	25	9	36
%		2.08%	0.30%	0.76%

## 4. Discussion

AST by the disc diffusion technique is a simple and reliable method that presents low-cost. This technique is the most common method to determine the susceptibility of antibiotics *in vitro* used worldwide, and it is considered the gold standard for detecting several resistance mechanisms [4,17]. However, the reading of the inhibition zones can be affected by the inherent visual error of the reader, emphasizing the need for standardization of the method. To address this, several automated systems have been developed and evaluated over the past decades to streamline and enhance the speed and reliability of AST interpretation [18–21]. Similarly, our institution developed an in-house automated disc diffusion reader named AntibiHórus, capable of reading and interpreting the inhibition zone diameters of the AST using a built-in camera and a user-friendly interface. AntibiHórus was projected with low-cost and widely available components, which facilitates implementation in resource-limited laboratories; an adaptable software pipeline that can be rapidly updated according to EUCAST breakpoint changes; and a compact and straightforward design that allows incorporation into routine workflows without the need for complex infrastructure.

In this study, we evaluated the ability of AntibiHórus to read inhibition zones by the disc diffusion methodology and interpret AST results. Thirty-nine bacterial species, including both Gram-negative and Gram-positive bacteria, were tested with 22 different antibiotics, resulting in 4,709 isolate-antibiotic combinations. The performance of AntibiHórus yielded excellent results, with only 0.76%, 0.30%, and 2.08% of MiE, ME, and VME, respectively. It is important to note that when excluding results in which the difference between the automated reading and the manual ruler measurement was 1 mm – an intrinsic variability of the disk diffusion method that also affects human interpretation – the VME rate would decrease to 1.5%, thereby meeting ISO performance criteria [16]. It is also worth emphasizing that the instrument allows manual adjustment of inhibition zone diameters. However, in the present study, we deliberately chose to evaluate the system’s performance without human intervention in order to more accurately assess its intrinsic analytical capability.

Noteworthy, the AntibiHórus software was designed to interpret halos according to EUCAST guidelines, and the breakpoints can be updated whenever necessary. Additionally, the system is designed to identify intrinsic resistance, which aids the operator in reviewing the results and contributes to more reliable and faster outcomes compared to conventional manual reading. Furthermore, the data generated by AntibiHórus can be saved for future reference and comparison purposes.

Based on the experimental results shown in this study, we conclude that the AntibiHórus system stands out for its excellent evaluation metrics and reliable performance. By streamlining and automating the analysis of inhibition zones, the software has demonstrated high accuracy, as evidenced by the strong correlation coefficient achieved compared to the conventional method. Its intuitive interface and adaptability to various antibiotic discs and microorganism species further enhance its usability, offering fast and accurate results. AntibiHórus proved to be a valuable tool for healthcare professionals and laboratories, assisting in clinical decision-making and contributing to the fight against antimicrobial resistance. This study was designed to evaluate the performance of an in-house automated disk-diffusion reader that has been implemented in our routine diagnostic laboratory. While the device was not initially conceived for broad distribution, we are available to collaborate with laboratories interested in developing or adapting similar equipment for local use. Such collaborations may foster independent validation and contribute to the dissemination of affordable automated AST solutions.

## Supporting information

S1 File Comparison between manual reading versus AntibiHórus – Template B. (XLSX)

S2 File Comparison between manual reading versus AntibiHórus – Template TU1. (XLSX)

S3 File Comparison between manual reading versus AntibiHórus – Template C +.
(XLSX)

S4 File Comparison between manual reading versus AntibiHórus – Template NF. (XLSX)

S5 File Comparison between manual reading versus AntibiHórus – Template TH. (XLSX)
